# A rare anastomosis between the root of common hepatic artery and proper hepatic artery: implications for pancreaticoduodenectomy

**DOI:** 10.1186/s40792-019-0746-y

**Published:** 2019-11-19

**Authors:** Takeshi Morinaga, Katsunori Imai, Keisuke Morita, Kenichiro Yamamoto, Satoshi Ikeshima, Kei Horino, Shinya Shimada, Hideo Baba

**Affiliations:** 1 Department of Surgery, Kumamoto General Hospital, Community Health Care Organization, Kumamoto, Japan; 2 Department of Gastroenterological Surgery, Graduate School of Life Sciences, Kumamoto University, 1-1-1 Honjo, Chuo-ku, Kumamoto, 860-8556 Japan

**Keywords:** Rare anastomosis, Pancreatic cancer, Pancreaticoduodenectomy, Arterial reconstruction

## Abstract

**Background:**

Hepatic artery anomalies are often observed, and the variations are wide-ranging. We herein report a case of pancreatic cancer involving the common hepatic artery (CHA) that was successfully treated with pancreaticoduodenectomy (PD) without arterial reconstruction, thanks to anastomosis between the root of CHA and proper hepatic artery (PHA), which is a very rare anastomotic site.

**Case presentation:**

A 78-year-old woman was referred to our department for the examination of a tumor in the pancreatic head. Contrast-enhanced computed tomography (CT) revealed a low-density tumor of 40 mm in diameter located in the pancreatic head. The involvement of the common hepatic artery (CHA), the root of the gastroduodenal artery (GDA), and portal vein was noted. Although such cases would usually require PD with arterial reconstruction of the CHA, it was thought that the hepatic arterial flow would be preserved by the anastomotic site between the root of the CHA and the PHA, even if the CHA was dissected without arterial reconstruction. PD with dissection of the CHA and PHA was safely completed without arterial reconstruction, and sufficient hepatic arterial flow was preserved through the anastomotic site between the CHA and PHA.

**Conclusion:**

We presented an extremely rare case of an anastomosis between the CHA and PHA in a patient with pancreatic cancer involving the CHA. Thanks to this anastomosis, surgical resection was successfully performed with sufficient hepatic arterial flow without arterial reconstruction.

## Introduction

The recent development of imaging modalities, such as three-dimensional computed tomography (CT) angiography, is helpful for better understanding vessel anomalies before surgery. Adequate knowledge of these variations would be of incredible help to the surgeon and interventional radiologist for avoiding unexpecting perioperative complications and unnecessary procedures [1–3]. Hepatic artery variations, including accessory right hepatic artery branching from the gastroduodenal artery, have previously been reported [4–9]; however, to our knowledge, there are no previous reports of anastomosis between the root of the common hepatic artery (CHA) and proper hepatic artery (PHA). We herein introduce a case in which pancreaticoduodenectomy (PD) was performed without arterial reconstruction, thanks to anastomosis between the root of the CHA and PHA.

## Case presentation

A 78-year-old woman was referred to our department for investigation of a tumor in the pancreatic head that was discovered upon a worsening of her diabetes. A physical examination revealed upper abdominal pain and jaundice. A laboratory analysis provided the following results: serum aspartate aminotransferase, 312 U/L (normal range, 13–30 U/L); alanine aminotransferase, 222 U/L (normal range, 10–42 U/L); lactate dehydrogenase, 266 U/L (normal range, 0–229 U/L); alkali-phosphatase, 3215 U/L (normal range, 0–359 U/L); γ-glutamyl transpeptidase, 835 U/L (normal range, 0–55 U/L); amylase, 12 U/L (normal range, 40–126 U/L); total bilirubin, 32.5 mg/dL (normal range, 0.4–1.5 mg/dL); direct bilirubin, 23.0 mg/dL (normal range, 0–0.2 mg/dL); albumin, 2.4 g/dL (normal range, 4.1–5.1 g/dL); prothrombin time (PT), 14.9 s (normal range, 11.0–15.2 s); activated partial thromboplastin time, 32.0 s (normal range, 25.2–35.2 s); hemoglobin A1c, 4.9% (normal range, 4.6–6.1%); and blood glucose, 166 mg/dL (normal range, 70–109 mg/dL). The serum level of CEA was normal, but the CA19–9 level was 297.3 U/mL (normal range, < 37 U/mL).

**Fig. 1:**
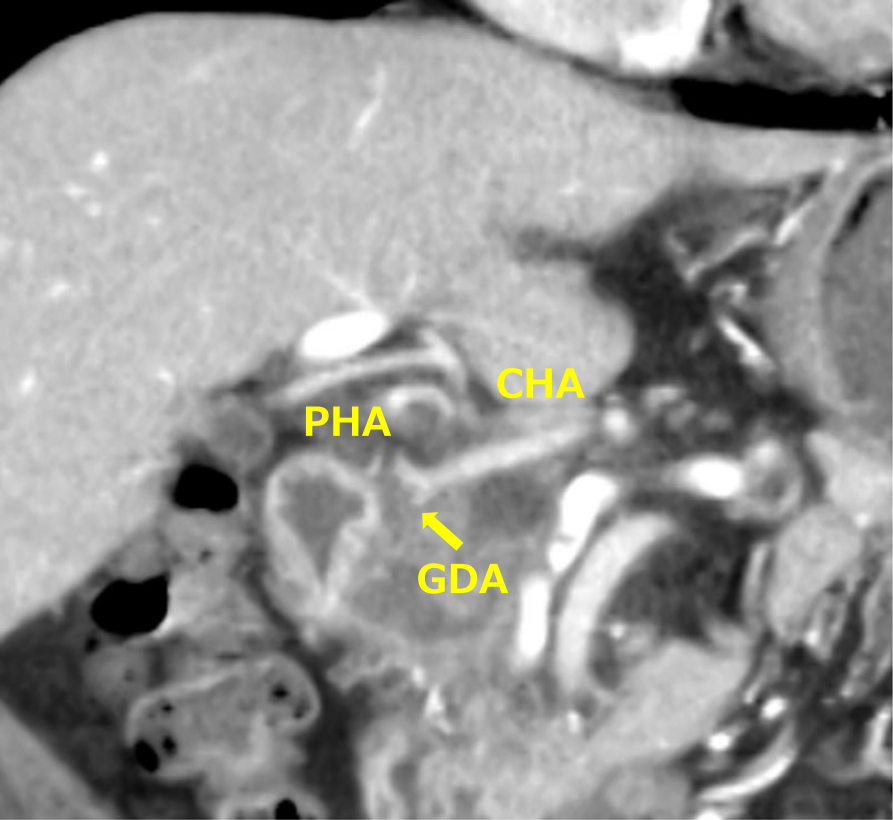
Contrast-enhanced CT revealed a low-density tumor in the pancreatic head, which was invading the CHA and the root of the GDA. CHA, common hepatic artery; PHA, proper hepatic artery; GDA, gastroduodenal artery

**Fig. 2:**
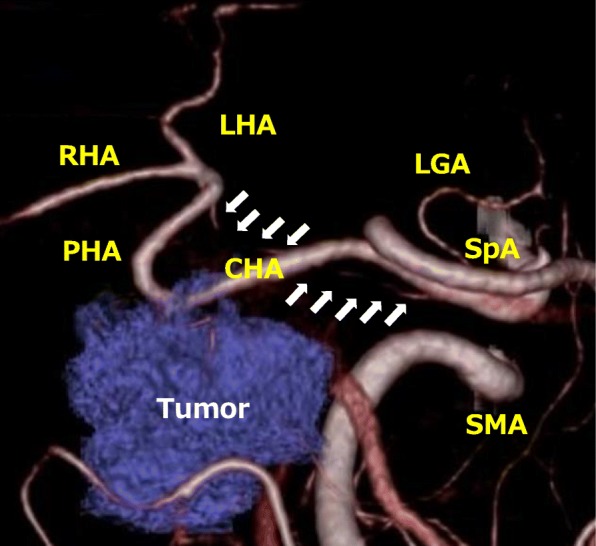
Three-dimensional CT angiography showed anastomosis between the root of the CHA and PHA (white arrow). RHA, right hepatic artery; LHA, left hepatic artery; LGA, left gastric artery; SpA, splenic artery; SMA, superior mesenteric artery

Contrast-enhanced CT revealed a low-density tumor of 40 mm in diameter located in the pancreatic head; involvement of the CHA, the root of the gastroduodenal artery (GDA), and the portal vein (PV) was noted (Fig. 1). The preoperative diagnosis was pancreatic head cancer with the involvement of the CHA and PV, T3N1M0 stage IIb (UICC seventh edition) [10]. Although such cases would usually require PD with arterial reconstruction of the CHA, three-dimensional CT angiography revealed a very rare arterial anomaly: anastomosis between the root of the CHA and PHA (Fig. 2). It was considered that the hepatic arterial flow could be preserved if the CHA was dissected without arterial reconstruction of the CHA; thus, we planned PD and PV resection and reconstruction, without arterial reconstruction of the CHA.

**Fig. 3:**
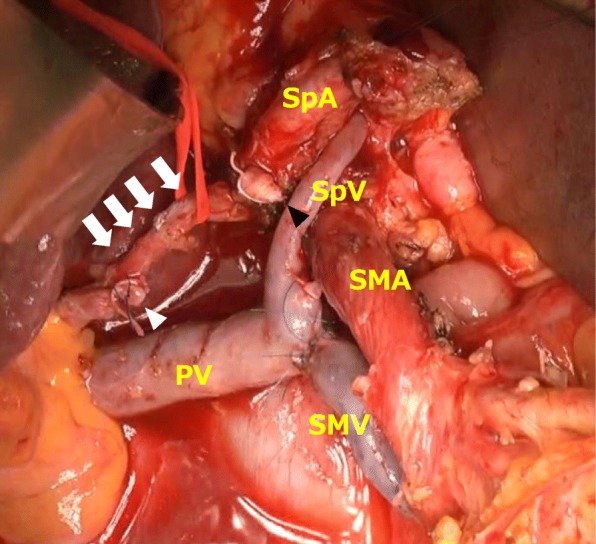
During the operation, anastomosis between the CHA and PHA was observed (white arrow). The anastomotic site became enlarged after dissection of the CHA. The black arrowhead shows the stump of the CHA. The white arrowhead shows the stump of the PHA. SpV, splenic vein; PV, portal vein; SMV, superior mesenteric vein

During the operation, the CHA and PHA were exposed and taped. After clamping both the CHA and PHA at the dissection line with a sufficient surgical margin, the anastomotic site between the root of the CHA and the PHA became enlarged and sufficient hepatic arterial flow, almost equivalent to that before vascular clamping, was confirmed by intraoperative pulse-wave Doppler ultrasonography. The CHA and PHA were dissected and PD was safely completed with sufficient hepatic arterial flow due to anastomosis between the CHA and PHA (Fig. 3).

Histologically, as expected before surgery, the pancreatic head cancer invaded the root of the gastroduodenal artery (GDA) and PV; however, cancer cells were not invading the lumen of the GDA. The final diagnosis was pancreatic head cancer with the involvement of the CHA and PV, T3N0M0 stage IIA (UICC seventh edition) [10].

**Fig. 4:**
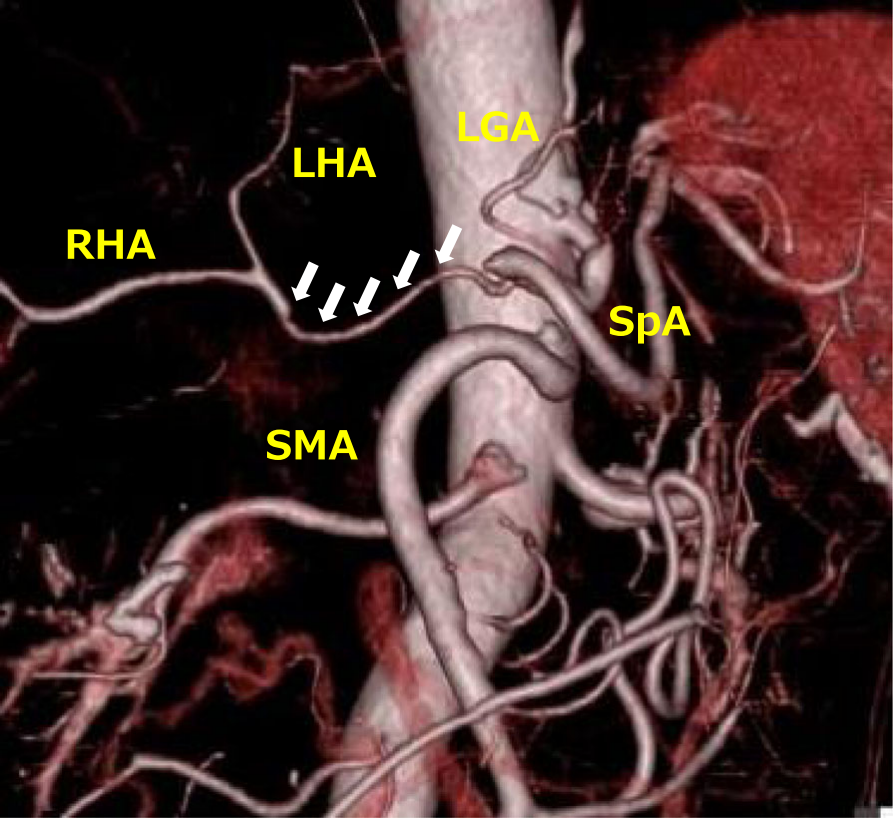
Follow-up CT angiography at 3 months after surgery demonstrated sufficient hepatic flow through the anastomotic site between the CHA and PHA (white arrow)

The patient did not develop any complications after surgery, including an impaired liver function or hepatic arterial flow-related complications such as biliary anastomotic leakage or stricture. Follow-up CT angiography at 3 months after surgery revealed sufficient hepatic flow through the anastomotic site between the CHA and PHA (Fig. 4). This patient developed recurrence at multiple sites in the liver 7 months after surgery and died of disease progression at 8 months after surgery.

## Discussion

Some previous studies have reported that hepatic artery anomalies are often observed and that the variation is wide-ranging [4–9]. The recent development of imaging modalities has enabled us to understand vessel anomalies preoperatively. A better understanding of the anatomy of vessel structures before surgery can help to avoid unexpected perioperative complications and unnecessary procedures [1–3]. Thus, adequate knowledge of these variations would be of incredible help to the surgeon and interventional radiologist [11].

Various hepatobiliary anomalies arise during the development process. During development, the coeliac axis is derived from the 10th ventral segmental artery and the superior mesenteric artery (SMA) is derived from the 13th segmental artery. The 11th and 12th segmental arteries normally regress [12, 13]. Variations occur during this development and regression of the ventral segmental arteries lead to multiple anomalies. Following normal development, the common hepatic artery usually arises as one of the three major branches of the coeliac axis. In adults, there are numerous anatomic variations of the hepatic arterial tree that result from the variable persistence of elements of the embryologic blood supply [14].

We always check the vessel structure anatomy before hepatobiliary surgery. In the present case, anastomosis between the root of the CHA and PHA was found. To the best of our knowledge, this is the first report of anastomosis between the root of the CHA and PHA that was identified prior to PD, although, it was unclear whether this developed congenitally or was acquired. Based on the observation that the pancreatic head cancer did not close the lumen of GDA histologically, it is considered that—in this case—anastomosis was congenital and arose in the development process.

In the present case, we considered that the hepatic arterial flow could be preserved if the CHA was dissected without arterial reconstruction due to anastomosis between the root of the CHA and PHA. After clamping both the CHA and PHA, sufficient hepatic arterial flow via this anastomotic site, which was almost equivalent to that before clamping, was confirmed by pulse-wave Doppler method on intraoperative ultrasonography. Although we were preparing for arterial reconstruction in the event that sufficient arterial flow could not be confirmed, the operation was successfully completed without arterial reconstruction, and the postoperative course was uneventful.

## Conclusion

We presented an extremely rare case of an anastomosis between the CHA and PHA in a patient with pancreatic cancer involving the CHA. Thanks to this anastomosis, hepatic arterial flow was sufficiently preserved without arterial reconstruction after PD. The importance of a preoperative understanding of vessel anomalies should be emphasized.

## Data Availability

The dataset supporting the conclusions of this article is included within the article.

## Abbreviations

CHA: Common hepatic artery
CT: Computed tomography
GDA: Gastroduodenal artery
PD: Pancreaticoduodenectomy
PHA: Proper hepatic artery
PV: Portal vein
